# Primary Hyper-aldosteronism in pregnancy: a case report with review of the literature

**DOI:** 10.1515/crpm-2025-0028

**Published:** 2026-05-13

**Authors:** Elie Hobeika, Dalia Malaeb, Dina Chamsy, Abdallah Adra, Nabil El Khoury, Anwar H. Nassar

**Affiliations:** Department of Obstetrics and Gynecology, Faculty of Medicine, American University of Beirut, Beirut, Lebanon; Department of Obstetrics and Gynecology, Kanad Hospital, Al Ain, Abu Dhabi, United Arab Emirates

**Keywords:** primary hyper-aldosteronism, hypertension, diagnosis, treatment

## Abstract

**Objectives:**

Primary hyperaldosteronism can present during pregnancy. Due to its rarity and to the physiological changes occurring during pregnancy, diagnosis might be delayed with potential complications imposed on the mother and newborn.

**Case presentation:**

We report a 46-years-old middle eastern patient with chronic hypertension, who conceived with assisted reproductive technologies and was diagnosed with PA. She was referred back to cardiology for re-evaluation of her resistant hypertension. Serum electrolytes showed low potassium, high aldosterone, and high renin to aldosterone ratio. Magnetic resonance imaging of the adrenal glands revealed a unilateral nodule in the left adrenal gland. The patient was maintained on three antihypertensives; unfortunately, her pregnancy was complicated by intrauterine growth restriction and preterm delivery for preeclampsia with severe features.

**Conclusions:**

Primary hyperaldosteronism is a rare complication of pregnancy that is challenging to diagnose. However, timely and accurate diagnosis are essential to initiate appropriate treatment to ensure optimal fetal and maternal outcomes.

## Introduction

Primary hyper-aldosteronism (PA) is a rare condition that occurs during pregnancy with very few cases reported in literature [1]. It impacts around 5–10 % of pregnancies around the globe [2]. The most common causes of this condition encompass bilateral adrenal hyperplasia and adrenal tumors producing aldosterone [3]. The ability of a clinician to properly diagnose and treat this condition in a timely manner is crucial to ensure optimal fetal and maternal outcomes [4]. While most reported cases of PA in pregnancy are either due to adrenal hyperplasia or unilateral tumors, the data on the disease course and outcomes vary considerably; thereby, most recommendations are based on data obtained from case reports [5]. Unfortunately, there might be a tendency to overlook causes of secondary hypertension in pregnant women, especially when the elevation in blood pressure occurs after 20 weeks of gestation or is associated with preeclampsia [6].

## Case presentation

We describe a case of a 46-years-old middle eastern G2P0A0L0E1 patient who conceived by assisted reproductive technologies, with a history of chronic hypertension. The patient had elevated blood pressure early in pregnancy, despite being maintained on 250 mg of Aldomet three times daily during her infertility treatment. Throughout her antenatal visit at 7 weeks of gestation, blood pressure was found to be high (145/105), regardless of increasing her Aldomet doses to 500 mg three times daily. She started on Labetalol 200 mg twice daily but continued to have symptomatic hypertension and reported experiencing headaches, orthostatic hypotension, and palpitations. Hence, she was referred to the cardiology team for re-evaluation of her resistant hypertension. Serum electrolytes showed a potassium level of 3.2 mmol/L, a high aldosterone level at 351 pg/mL (normal range: 30–146 pg/mL), and a high renin to aldosterone ratio of 195 (normal: <64), whereas adrenaline, noradrenaline, and dopamine levels were normal. Ultrasound of the kidneys excluded the presence of renal artery stenosis. Nevertheless, magnetic resonance imaging (MRI) of the adrenal glands revealed a 0.4 cm nodule in the left adrenal gland, suggestive of an adrenal adenoma.

The patient was diagnosed with PA, and Labetalol was discontinued. She was kept on three antihypertensive medications on a daily basis (Bisoprolol 5 mg, Aldomet 500 mg three times, and Nifedipine Long Acting 90 mg) until 12 weeks of gestation. Aldomet was then replaced by Spironolactone (titrated up to 100 mg daily). She had a close antenatal follow-up with weekly fetal non-stress test and biophysical profile between 28 and 32 weeks, followed by twice weekly testing. In addition, preeclampsia work-up (involving CBC, creatinine, ALT, AST, uric acid, LDH and urine analysis to check on proteinuria) and serum potassium were carried out on a weekly basis. Despite her compliance with the medications, her blood pressure readings continued to be labile. She received a course of antenatal corticosteroids for fetal lung maturity at 31 weeks in anticipation of preterm delivery. Growth scans showed intrauterine fetal growth restriction (IUGR), with an estimated fetal weight at less than 10th percentile.

At 34 weeks of gestation, the patient was admitted to the delivery suite for uncontrolled blood pressure (systolic readings: 170–180 mmHg), associated with headache refractory to Acetaminophen. She started on Labetalol drip as her blood pressure was refractory to Labetalol. With a presumptive diagnosis of preeclampsia accompanying severe features, magnesium sulfate was started for seizure prophylaxis. One rescue dose of steroids was given for lung maturity; stabilizing her blood pressure and improving her symptoms. Twelve hours following steroid injection, a primary cesarean delivery was performed for two previous myomectomies. The patient delivered a female infant weighing 1,812 g with Apgar scores of 8 and 9 at 1 and 5 min respectively. She was monitored for 24 h while being sustained on magnesium sulfate. Cardiology team maintained her daily on Bisoprolol 5 mg, Spironolactone 50 mg, and Losartan 80 mg.

The patient was discharged home on day 4 postpartum, with controlled blood pressure, and a plan to follow up with the cardiology and endocrinology teams.

## Discussion

While PA is rarely diagnosed in women of childbearing age, our patient is 46-years-old, close to the mean age at which PA is usually diagnosed [4]. The manifestation of hypertension was evident early in gestation, which is yet another distinguishing feature of secondary causes of hypertension [7]. The cause of PA in our case originated from a unilateral adrenal tumor. The patient’s hypertension was managed with antihypertensives combined with Spironolactone which had been used in similar cases [4], 8], but in contrast to other ones [9]. Evidence from literature shows that the use of Eplerenone is a better choice in light of its safety profile during pregnancy [10]; nonetheless, it wasn’t available in our case. Furthermore, surgical treatment of the tumor was not entertained as severe hypertension refractory to medications was not encountered [11].

Diagnosing PA during pregnancy is very challenging [6]. The presence of severe hypertension associated with hypokalemia should prompt screening for PA. Relying solely on elevated levels of circulating aldosterone is not enough for diagnosis since moderate elevation in aldosterone levels is considered a physiological change during pregnancy [12]. Rather, the presence of elevated aldosterone with a suppressed serum renin concentration and an elevated aldosterone to renin ratio is required to confirm the diagnosis [4]. Moreover, the American Heart Association states that hypokalemia may not be present in all pregnant women with PA [13]. This has been explained by the effects of the high levels of circulating progesterone which inhibit the effect of aldosterone on potassium levels [14]. Consequently, a significant number of PA cases are diagnosed postpartum, when the levels of progesterone start dropping.

Management of PA in pregnancy can be medical or surgical. Medically, options include mineralocorticoid receptor antagonists such as Spironolactone (category C) and Eplerenone (category B) [4]. However, fear of using Spironolactone during pregnancy emerges because of its anti-androgenic effects in male fetuses, and potential role in intrauterine growth restriction (IUGR) [15]. Unequivocally, Eplerenone seems to be a safer alternative [8], due to its selective action discarding any anti-androgenic concerns [16]. Surgically, resection of an adrenal adenoma is performed when hypertension and hypokalemia become refractory to medical therapy, and it is preferably done laparoscopically in the second trimester [11], 17].
